# Reactive oxygen species do not contribute to ObgE*-mediated programmed cell death

**DOI:** 10.1038/srep33723

**Published:** 2016-09-19

**Authors:** Liselot Dewachter, Pauline Herpels, Natalie Verstraeten, Maarten Fauvart, Jan Michiels

**Affiliations:** 1 Centre of Microbial and Plant Genetics, KU Leuven - University of Leuven, 3001 Leuven, Belgium; 2 Department of Life Science Technologies, Smart Systems and Emerging Technologies Unit, imec, 3001 Leuven, Belgium

## Abstract

Programmed cell death (PCD) in bacteria is considered an important target for developing novel antimicrobials. Development of PCD-specific therapies requires a deeper understanding of what drives this process. We recently discovered a new mode of PCD in *Escherichia coli* that is triggered by expression of a mutant isoform of the essential ObgE protein, ObgE*. Our previous findings demonstrate that ObgE*-mediated cell death shares key characteristics with apoptosis in eukaryotic cells. It is well-known that reactive oxygen species (ROS) are formed during PCD in eukaryotes and play a pivotal role as signaling molecules in the progression of apoptosis. Therefore, we explored a possible role for ROS in bacterial killing by ObgE*. Using fluorescent probes and genetic reporters, we found that expression of ObgE* induces formation of ROS. Neutralizing ROS by chemical scavenging or by overproduction of ROS-neutralizing enzymes did not influence toxicity of ObgE*. Moreover, expression of ObgE* under anaerobic conditions proved to be as detrimental to bacterial viability as expression under aerobic conditions. In conclusion, ROS are byproducts of ObgE* expression that do not play a role in the execution or progression of ObgE*-mediated PCD. Targeted therapies should therefore look to exploit other aspects of ObgE*-mediated PCD.

The study of bacterial programmed cell death (PCD) is a relatively new but promising research area that can lead to the development of innovative antibacterial compounds that artificially induce PCD in bacteria^1^. We previously discovered a new PCD mechanism in *E. coli* that is induced by a toxic isoform of the essential protein, ObgE^2^. ObgE is a GTPase involved in many key cellular processes such as ribosome assembly, the stringent response, chromosome segregation and persistence^3^,^4^,^5^,^6^,^7^,^8^. The introduction of the K268I mutation in ObgE (referred to as ObgE*) renders this protein highly toxic to the cell. Upon expression of ObgE*, cells rapidly die through an ordered PCD mechanism. Indeed, ObgE*-mediated toxicity is associated with many markers of PCD, such as chromosome condensation, DNA fragmentation and exposure of phosphatidylserine on the cell surface. The phenotypic changes that occur during ObgE*-mediated PCD greatly resemble the hallmarks of eukaryotic apoptosis^2^,^9^.

Reactive oxygen species (ROS) such as superoxide anions (O_2_^•−^), hydrogen peroxide (H_2_O_2_) and hydroxyl radicals (OH^•^) are known to play a physiological role in apoptosis. ROS are formed when O_2_ is incompletely reduced. These molecules are highly reactive and can confer damage to lipids, proteins and DNA. Life in aerobic environments is inevitably associated with the formation of ROS. Under normal circumstances, organisms can cope with ROS and efficiently neutralize these damaging agents or repair the inflicted damage. However, when the concentration of ROS inside the cells becomes overwhelming and defense mechanisms fail, cells may succumb to this oxidative stress and die^10^,^11^,^12^,^13^.

Rather than being the executioners of cell death, ROS can also function as secondary messengers in cell death pathways, as is the case for eukaryotic apoptosis^10^,^11^,^14^,^15^,^16^. This PCD mechanism can be triggered by external or internal signals that respectively activate the extrinsic or intrinsic pathway of apoptosis. Intracellular ROS can directly trigger the intrinsic pathway and can also modulate this pathway at later stages^10^,^11^,^14^. For example, ROS can regulate the activity of BCL-2 family members^14^, influence the release of cytochrome *c* from mitochondria^11^,^14^ and directly alter the activity of caspases^10^,^11^,^14^. Moreover, ROS are also involved in the extrinsic apoptosis signaling pathway^10^,^11^,^15^. For example, stimulation of extracellular death receptors leads to the formation of ROS that subsequently act as secondary messengers in this apoptotic pathway^10^.

Since ROS are implicated in the onset and progression of eukaryotic apoptosis^10^,^11^,^14^,^15^,^16^, a PCD pathway that shares many characteristics with ObgE*-mediated PCD, and because several bacterial PCD mechanisms are also associated with the formation of ROS^17^,^18^,^19^,^20^, we investigated whether ROS are produced during ObgE*-mediated PCD. Furthermore, we questioned whether ROS contribute to killing either directly, as is proposed for the killing mechanism of bactericidal antibiotics^21^, or indirectly by functioning as a signaling molecule important for the progression of ObgE*-mediated PCD. Our results demonstrate that ROS are formed upon expression of ObgE*, although they play no role in the execution or progression of ObgE*-mediated PCD.

## Results

### ObgE* increases intracellular ROS concentrations

**Figure 1:**
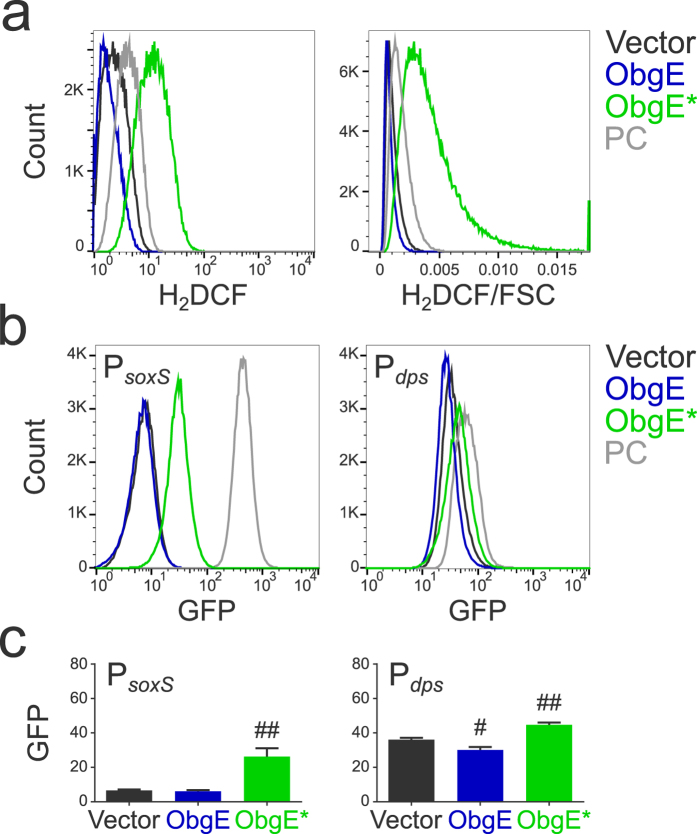
ObgE* increases intracellular ROS concentrations. (**a**) Cultures of *E. coli* pBAD33, *E. coli* pBAD33-*obgE* or *E. coli* pBAD33-obgE* were stained with the redox-sensitive probe H_2_DCFDA that can be oxidized to form the fluorescent H_2_DCF. ObgE* expression increases H_2_DCF fluorescence, even when the signal is corrected for cell size, and thus causes oxidative stress. PC = Positive Control, *E. coli* pBAD33 + 100 μM H_2_O_2_. (**b**) ObgE* activates GFP expression from the ROS-responsive promoters P_*soxS*_ and P_*dps*_ and therefore leads to increased ROS concentrations. PC = Positive Control, *E. coli* pBAD33 + 20 mM PQ for P_*soxS*_ and *E. coli* pBAD33 + 100 μM H_2_O_2_ for P_*dps*_. Representative results of three repeats are shown. (**c**) Average GFP expression from the ROS-responsive promoters P_*soxS*_ and P_*dps*_ shows that ObgE* expression leads to increased ROS concentrations. Averages of three repeats are shown. Error bars represent the standard error of the mean, n = 3. Student’s t test: ^#^p-value < 0.05, ^##^p-value < 0.01 in comparison with the vector control.

Because ROS are formed during eukaryotic apoptosis and several bacterial PCD mechanisms^10^,^11^,^14^,^15^,^16^,^17^,^18^,^19^, we investigated whether a surge in ROS concentration also occurs during ObgE*-mediated PCD in *E. coli*. Two different approaches were exploited. The first approach relies on the molecule H_2_DCFDA (2,7-dichlorodihydrofluorescein diacetate), a non-fluorescent precursor that can be oxidized by several ROS to form the fluorescent probe H_2_DCF (2,7-dichlorofluorescein). H_2_DCF fluorescence can thus be used as a measure for general oxidative stress^22^. It was shown previously that fluorescence of such probes correlates linearly with cell size independently of the amount of ROS present^23^. To compensate for this effect, fluorescence was corrected for cell size by dividing the fluorescent signal by the forward scatter (FSC). Furthermore, H_2_DCF fluorescence is also influenced by pH^24^. We therefore confirmed that pH is constant in all conditions tested (data not shown). Both the H_2_DCF signal and the H_2_DCF/FSC ratio are shown in Fig. 1a. As a positive control H_2_O_2_ was added. These results indicate that expression of ObgE* indeed causes oxidative stress.

Although H_2_DCF fluorescence was normalized to account for variations in cell length and pH was constant, other issues with redox-sensitive fluorescent dyes exist^13^. A more reliable way of measuring intracellular ROS concentration makes use of the cell’s innate detection mechanisms. We therefore used a plasmid-based system where the expression of GFP is controlled by one of two different redox-sensitive promoters, P_*soxS*_ and P_*dps*_^25^. P_*soxS*_, the promoter of the *soxS* gene, is activated by SoxR in the presence of a variety of oxidants such as redox-cycling agents and possibly also O_2_^•− ^26. P_*dps*_ is the promoter of the *dps* gene that can be activated by OxyR. OxyR induces expression of *dps* upon exposure to H_2_O_2_^27^. Paraquat (PQ) and H_2_O_2_ were used as a positive control for the activation of P_*soxS*_ and P_*dps*_, respectively, and GFP fluorescence was detected as a measure for either general oxidative stress/O_2_^•−^ or H_2_O_2_. Results are consistent with data obtained with the H_2_DCFDA probe and show that ObgE* leads to an increase in oxidative stress and H_2_O_2_. This effect could not be seen when wild-type ObgE was overexpressed. In fact, expression of ObgE even leads to a significant decrease in intracellular H_2_O_2_ concentration compared to the vector control (Fig. 1b,c).

Taken together, our data clearly demonstrate that intracellular ROS levels increase upon ObgE* expression, suggesting a possible role for ROS in ObgE*-mediated cell death.

### ROS scavengers cannot rescue cells from ObgE*

**Figure 2:**
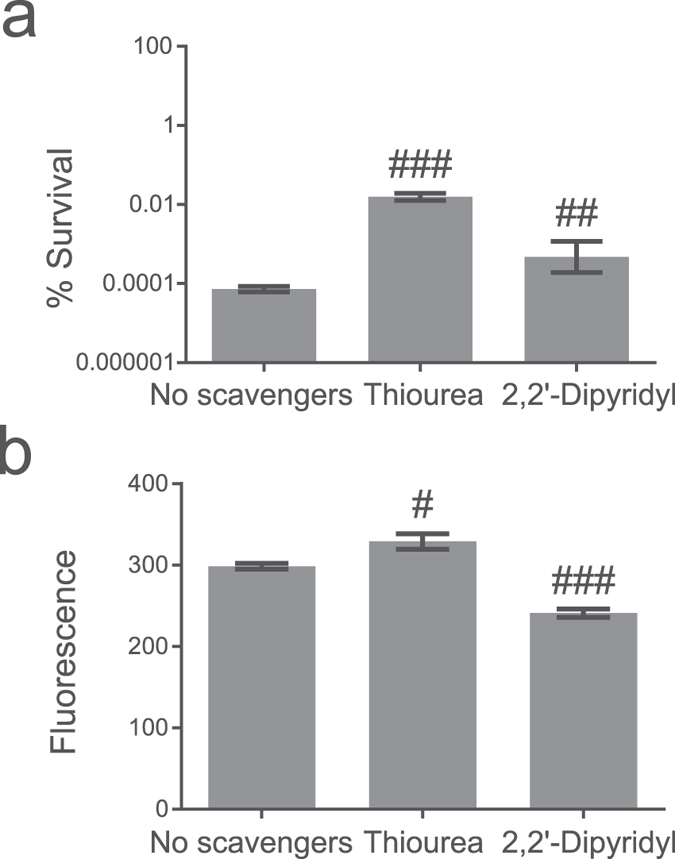
Addition of thiourea or 2,2′-dipyridyl causes a possibly non-specific increase in survival upon ObgE* expression. Exponential-phase cultures of *E. coli* pBAD33, *E. coli* pBAD33-*obgE* or *E. coli* pBAD33-obgE* were induced with 0.2% arabinose for 2 hours and thiourea or 2,2′-dipyridyl was simultaneously added to the culture. (**a**) Addition of thiourea or 2,2′-dipyridyl increases survival in the presence of ObgE*. (**b**) The expression level of ObgE* was determined by using a fluorescent fusion of ObgE* with Venus. ObgE*-Venus expression is lowered in the presence of 2,2′-dipyridyl. Error bars represent the standard error of the mean, n = 3. Student’s t test: ^#^p-value < 0.05, ^##^p-value < 0.01, ^###^p-value < 0.001 in comparison with ‘No scavengers’.

**Figure 3:**
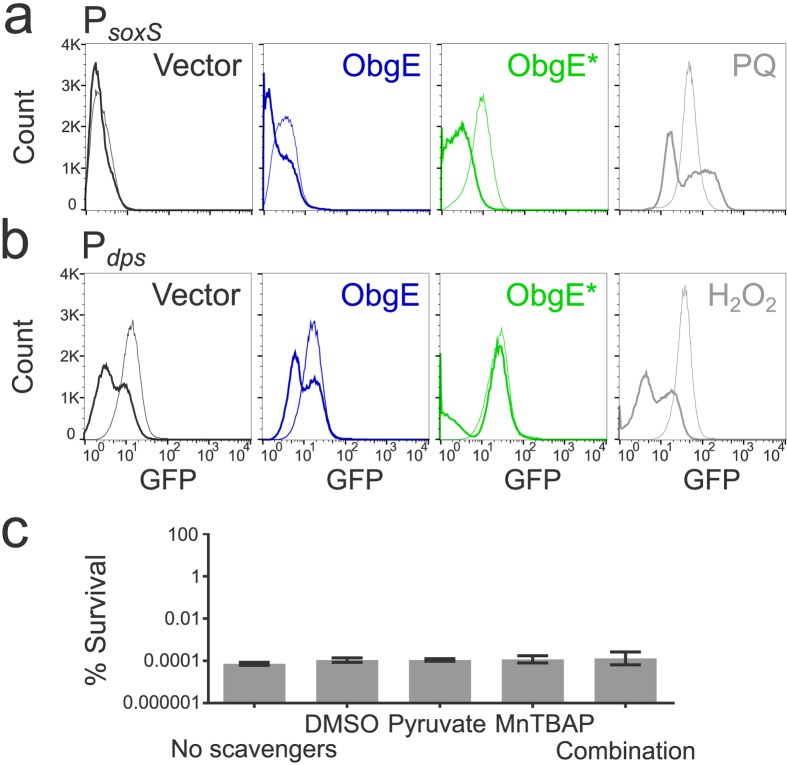
Scavengers lower ROS concentrations but do not influence ObgE*-mediated toxicity. Exponential-phase cultures of *E. coli* pBAD33, *E. coli* pBAD33-*obgE* or *E. coli* pBAD33-obgE* were induced with 0.2% arabinose for 2 hours and ROS-scavengers DMSO, sodium pyruvate and MnTBAP were simultaneously added to the culture. (**a**) The combination of ROS scavengers lowers the activity of the redox-sensitive promoter P_*soxS*_. As a positive control 20 mM paraquat (PQ) was used. Thick lines represent samples to which scavengers were added. (**b**) The combination of ROS scavengers lowers the activity of the H_2_O_2_-responsive promoter P_*dps*_. As a positive control 100 μM H_2_O_2_ was used. Thick lines represent samples to which scavengers were added. (**c**) The combination of ROS scavengers, or of each scavenger separately, has no effect on survival upon expression of ObgE*. Error bars represent the standard error of the mean, n = 4. Student’s t test: not significant (α = 0.05) in comparison with ‘No scavengers’.

Several methods were used to determine if ROS that are formed during ObgE* expression are important for the progression and/or execution of ObgE*-mediated cell death. First, the effect of ROS scavengers on toxicity was investigated. Research on the role of ROS in bacterial killing often makes use of the hydroxyl radical scavenger thiourea and the iron chelator 2,2′-dipyridyl that can prevent OH^•^ production by the Fenton reaction^21^,^28^. The effect of both compounds on ObgE*-mediated cell death was quantified (Fig. 2a). At the same time, the expression level of ObgE* was measured by expressing the protein as a fusion with Venus and measuring fluorescence (Fig. 2b). Results indicate that both compounds significantly increase survival. However, Fig. 2b shows that the expression level of ObgE* is lowered by the addition of 2,2′-dipyridyl, which can explain the observed increase in survival in the presence of this compound. In contrast, addition of thiourea did not affect ObgE* expression, indicating a possible role for ROS in toxicity of ObgE*. Both scavengers however, are known to cause non-specific side effects; protective effects in anaerobic conditions were previously detected and both compounds also have a negative effect on bacterial growth^29^,^30^. To circumvent these issues, three additional scavenging compounds with different specificities were selected; the superoxide dismutase mimic MnTBAP (manganese (III) tetrakis (4-benzoic acid) porphyrin) was added to neutralize O_2_^•− ^31, H_2_O_2_ was targeted by pyruvate^32^ and DMSO was selected as a scavenger for the highly deleterious OH^• ^16,18,33. Additionally, a combination of these three scavengers was used to prevent potential compensation of scavenging by elevated levels of non-target ROS^16^. The potency of this combination of scavengers to lower the intracellular ROS concentration was tested by assessing the effect on the activity of the P_*soxS*_ (Fig. 3a) and P_*dps*_ (Fig. 3b) promoters. This combination of compounds is indeed capable of lowering ROS concentration in at least part of the population. Moreover, the selected scavengers have a positive effect on survival in the presence of oxidative stress (Supplementary Fig. S1). These results strongly suggest that the combination of DMSO, pyruvate and MnTBAP is capable of protecting cells from ROS by lowering the intracellular concentration of these compounds. However, addition of each scavenger separately or their combination does not have any effect on survival in the presence of ObgE* (Fig. 3c). In conclusion, these data demonstrate that ROS are not major contributors to ObgE*-mediated cell death.

### ROS-neutralizing enzymes do not influence ObgE*-mediated PCD

**Figure 4:**
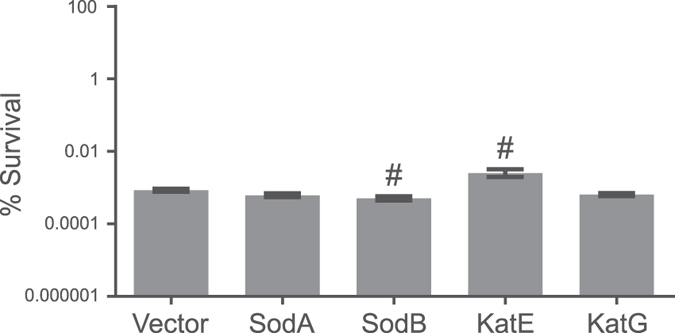
Overexpression of ROS-neutralizing enzymes cannot rescue cells from ObgE*. Cultures of *E. coli* with pQE80(Km) (vector control) or pQE80(Km)-*sodA*/*sodB*/*katE*/*katG* and pBAD33-*obgE* or pBAD33-obgE* were grown until exponential phase in the presence of 0.1 mM IPTG and then induced with 0.2% arabinose. The percentage of surviving cells was determined two hours after induction. No consistent increase in survival can be seen upon overexpression of ROS-neutralizing enzymes. Error bars represent the standard error of the mean, n = 4. Student’s t test: ^#^p-value < 0.05 in comparison to the vector control.

Another way to neutralize ROS is through overexpression of protective enzymes. While the superoxide dismutases SodA and SodB detoxify O_2_^•−^ in *E. coli*, intracellular H_2_O_2_ can be catalytically removed by the catalases KatE and KatG^34^. We therefore determined the effect of overexpression of each of these four enzymes on ObgE*-mediated toxicity. Results are shown in Fig. 4. In comparison with the vector control where no protective enzyme is overexpressed, expression of SodA or KatG does not alter survival in the presence of ObgE*. Some very mild though statistically significant changes in survival were detected when either SodB or KatE are overexpressed. These changes however, point in opposite directions; SodB causes a minor decrease in survival while KatE overexpression leads to a small increase. In addition, overexpression of a similar enzyme with the same function, either SodA or KatG, did not influence toxicity. We therefore conclude that the observed differences do not reflect an important role for ROS in ObgE*-mediated toxicity and that the overproduction of ROS-neutralizing enzymes cannot rescue cells from ObgE*.

### ObgE*-mediated PCD is unaltered in anaerobic conditions

**Figure 5:**
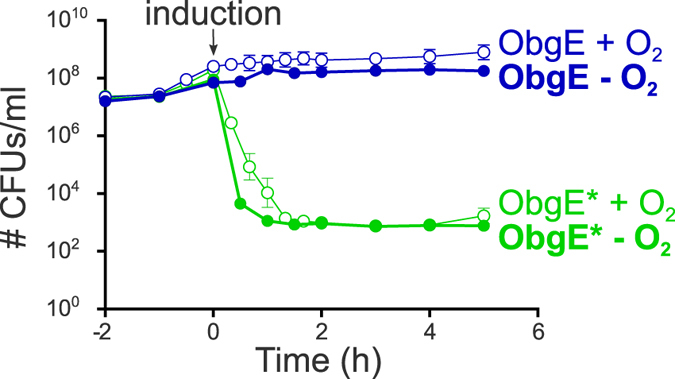
ROS play no role in ObgE*-mediated cell death. Cultures of *E. coli* pBAD33-*obgE* or *E. coli* pBAD33-obgE* were grown in the presence or absence of oxygen and expression of ObgE or ObgE* was induced with 0.2% arabinose at OD_595 nm_ 0.4. The change in number of viable cells was followed for 2 hours before and 5 hours after induction. Error bars represent the standard error of the mean, n = 3.

Scavenging ROS by the addition of chemical compounds or by overexpression of ROS-neutralizing enzymes indicates that ROS are not major contributors to ObgE*-mediated cell death. However, the possibility remains that small amounts of ROS function as signaling molecules necessary for the onset and/or progression of ObgE*-mediated PCD. The addition of scavengers or overexpression of ROS-neutralizing enzymes might then be insufficient to lower the level of ROS below the concentration that is needed to perform its signaling function. To rule out this possibility, ObgE*-mediated cell death was investigated in a strictly anaerobic environment where no ROS can be formed. Time kill curves were generated in the presence and absence of oxygen and are shown in Fig. 5. ObgE* expression in the absence of oxygen, and thus the absence of ROS, leads to the same baseline level of surviving cells as it does in aerobic conditions. This baseline however, is reached slightly faster when operating under anaerobic conditions. We believe this deviation is due to technical differences in working procedures. When operating under anaerobic conditions the workspace also functioned as incubator and therefore samples were never taken out of the incubator and exposed to lower temperatures as was the case for samples grown under aerobic conditions. Exposure to lower temperatures can slow down metabolism and ObgE* expression, possibly explaining the slightly slower killing kinetics under aerobic conditions.

Overall, this experiment shows that toxicity of ObgE* is not influenced by the absence of oxygen and presents convincing evidence that ROS that are formed during ObgE* expression do not play any role in this cell death mechanism; they do not contribute directly to bacterial killing, nor function as signaling molecules necessary for the progression of ObgE*-mediated PCD.

## Discussion

The molecular mechanisms of bacterial PCD are incompletely understood. More research is necessary to pinpoint important processes and signaling pathways involved in bacterial PCD. Because ROS are important signaling molecules in the progression of apoptosis^10^,^11^,^14^,^15^,^16^, the most important PCD mechanism in multicellular eukaryotes, we investigated whether ROS also play a role during a specific bacterial PCD pathway in *E. coli*. This pathway is triggered by expression of ObgE*, a mutant form of the essential GTPase ObgE^2^.

Using fluorescent probes and genetic reporters, we confirmed that ObgE* expression leads to the formation of ROS. The redox-sensitive probe H_2_DCFDA is oxidized in the presence of ObgE*, indicating that ObgE* expression causes oxidative stress. Since many studies report non-specific fluorescence increases of redox-sensitive probes, confirmation of these results by other means is necessary^13^,^23^. A highly reliable way to determine the presence of intracellular ROS is to use the cell’s innate detection mechanisms and check the activation of redox-sensitive or ROS-responsive promoters, such as P_*soxS*_ and P_*dps*_. The activity of both promoters is significantly upregulated in the presence of ObgE*, confirming results obtained with H_2_DCFDA. Remarkably, expression of the wildtype ObgE protein lowers the activity of P_*dps*_ and thus decreases the intracellular H_2_O_2_ concentration. How and why ObgE can decrease the level of H_2_O_2_ is currently not understood.

Although ROS can inflict damage to the cell, the concentration of ROS formed by ObgE* expression appears to be insufficient to directly contribute to cell death. The addition of the ROS-scavenging compounds DMSO, pyruvate and/or MnTBAP has no influence on survival even though this mixture of scavengers lowers intracellular ROS concentrations and protects cells from oxidative stress. Moreover, expression of ROS-neutralizing enzymes does not lead to a consistent increase in survival. Additionally, experiments in strictly anaerobic conditions show that ObgE*-mediated toxicity is not influenced by the presence or absence of oxygen. These experiments show that ROS do not directly contribute to ObgE*-mediated cell death. However, conflicting results were obtained when scavengers 2,2′-dipyridyl or thiourea were used. Addition of these compounds increased survival upon induction of ObgE*. We therefore investigated whether 2,2′-dipyridyl and thiourea could have a ROS-independent protective effect on survival, for example by lowering ObgE* expression levels. We were indeed able to show that 2,2′-dipyridyl lowers ObgE* concentration, explaining the protective effect of this compound. 2,2′-dipyridyl greatly influences bacterial metabolism by causing iron limitation^13^,^35^. The resulting reduced bacterial activity likely is the cause of decreased ObgE* expression. When the OH^•^ scavenger thiourea was added to the culture, no decrease in expression level could be detected. We even detected a small increase in the level of ObgE*. Nevertheless, addition of thiourea was associated with a decrease in ObgE*-mediated toxicity. However, since all other experiments performed argue against a role for ROS in cell death, we conclude that the increased survival in the presence of thiourea must be due to some non-specific effect other than lowering the ObgE* expression level. A ROS-independent protective effect for thiourea was indeed described previously, since it was shown that thiourea can also increase survival upon treatment with ampicillin in an anaerobic environment^29^,^30^. A protective effect of 2,2′-dipyridyl in anoxic conditions was found as well^29^. Overall, we strongly believe that results obtained with thiourea and 2,2′-dipyridyl should be validated by other scavengers, for example DMSO, pyruvate and MnTBAP, and complemented with additional tests such as those performed here. The overwhelming majority of these tests show that ROS are not the major executioners of ObgE*-mediated cell death. This is consistent with a recent estimation that even if 45% of the electron flow in the respiratory chain is directed at the production of H_2_O_2_, this would be insufficient to have a bactericidal effect^13^. ROS could potentially contribute to the bactericidal effect of ObgE* if protective systems would simultaneously be downregulated^13^. Proteomic analysis of bacteria expressing ObgE* showed, however, that this is not the case and that some ROS-neutralizing enzymes are actually upregulated upon ObgE*-expression (our own unpublished data). ROS are therefore not the direct cause of ObgE*-mediated bacterial cell death.

During eukaryotic apoptosis, ROS can act as signaling molecules that modulate the apoptotic response^10^,^11^,^14^,^15^,^16^. Because of the physiological similarities between eukaryotic apoptosis and the PCD pathway triggered by ObgE*, we wondered if ROS likewise perform a signaling function during ObgE*-mediated PCD. Since neither modulation of intracellular ROS levels nor expression of ObgE* in anaerobic environments can influence toxicity, we conclude that ROS do not perform an essential signaling function during ObgE*-mediated PCD.

In conclusion, we have shown that ROS are formed when ObgE* is expressed in *E. coli*. These ROS however, do not play any role in the execution or regulation of this cell death pathway and appear to be merely byproducts of ObgE* expression. Since the most likely source of ROS formation is the electron transport chain, we hypothesize that ObgE* leads to malfunctioning of the respiratory chain and leakage of electrons at intermediary positions. However, since ROS are not important for ObgE*-mediated PCD, our current research is focused on investigating other possible causes of bacterial killing by ObgE*. Recently, a novel cell death pathway in *E. coli* was described that depends on the disruption of lipid homeostasis^36^. Although many of the features of this cell death pathway are not present in ObgE*-mediated cell death, remarkable similarities between both exist. For example, both cell death pathways are associated with the formation of membrane blebs and result in the loss of membrane integrity. ObgE* might therefore exert its toxic effect through disruption of lipid homeostasis.

Further insight into the mechanism underlying ObgE*-mediated PCD might prove valuable in the search for novel antimicrobials. The existence of PCD in bacteria implicates that bacterial cell death can be self-inflicted by activation of a cellular suicide mechanism. Uncovering the pathways leading to this type of cell death should allow us to control these processes and use them to our own advantage. Artificial induction of bacterial PCD could then be employed to combat bacterial infections and thus lead to the development of new classes of antibiotics. This alternative approach would give us an advantage in the arms race against the development of resistance. Since the essential GTPase Obg is highly conserved in bacteria^8^, ObgE*-mediated PCD may be a prime target for the development of novel broad-spectrum antibacterial therapies.

## Methods

### Strains, plasmids and growth conditions

The *E. coli* BW25113 strain was used throughout this work. To investigate ObgE*-mediated effects, strains were transformed with pBAD33, pBAD33-*obgE* or pBAD33-obgE*. pBAD33-*obgE* was constructed by amplifying the *obgE* gene from pBAD/His A-*obgE*^7^ using primers CACCGAGCTCAGGAGGAATTAACCATGAAGTTTGTTGATGAA and GATCAAGCTTTTAAC GCTTGTAAAT. The resulting fragment was digested using SacI and HindIII and ligated into pBAD33^37^. pBAD33-obgE* was then constructed by site-specific mutagenesis using the QuickChange Site-Directed Mutagenesis Kit (Stratagene) with pBAD33-*obgE*. Primers used to introduce the K268I mutation are CTGGAAATATACAGCCAGGATCTG and CTGGCTGTATATTTCCAGCTCG. For all tests, overnight cultures were diluted 100 times in lysogeny broth (LB) containing the appropriate antibiotics and incubated at 37 °C with continuous shaking at 200 rpm. When the OD_595 nm_ reached 0.4, bacterial cultures were induced with arabinose (0.2% w/v) for 2 hours under the same conditions. For colony counts, serial dilutions were prepared in 10 mM MgSO_4_ and plated on medium containing 1.5% agar. The percentage survival was calculated by dividing the number of colony forming units (CFUs) per ml obtained after ObgE* overexpression by the number of CFUs per ml after ObgE expression. To determine the expression level of ObgE*, *E. coli* BW25113 pBAD33-obgE*-venus was used and Venus fluorescence was measured by flow cytometry. For the construction of pBAD33-obgE*-*venus*, a XhoI-BlgII fragment from pBAD/His A-obgE* was cloned into pBAD/His A-*obgE*-*venus*^7^. Subsequently, obgE*-*venus* was amplified with primers CACCGGTACCCACCAGGAGGAATTAACCATGAAGTTTGTTGATGAAGCATCG and AGCCAAGCTTCGAATTCTTA. The resulting fragment was digested using KpnI-HF and HindIII-HF and ligated into pBAD33. For detection of ROS by P_*soxS*_ or P_*dps*_ the plasmids pZE1-P*soxS*-GFP or pZE1-P*dps*-GFP were used, respectively^25^. SodA, SodB, KatE and KatG were overexpressed from pQE80(Km)^38^ by induction with IPTG (0.1 mM) at the time of dilution.

### ROS detection by H_2_DCFDA

Two hours after induction of ObgE or ObgE*, 1 ml of each culture was washed and dissolved in PBS. As a positive control, H_2_O_2_ (100 μM) was then added to a strain carrying the empty pBAD33 vector. H_2_DCFDA (10 μM) was added and tubes were incubated 30 min in the dark at room temperature. Fluorescence was measured using a BD Influx cell sorter equipped with a 488 nm laser and standard filter sets.

### ROS detection by redox-sensitive promoters

GFP fluorescence of *E. coli* with pBAD33, pBAD33-*obgE* or pBAD33-obgE* in combination with pZE1-P*soxS*-GFP or pZE1-P*dps*-GFP was measured by flow cytometry two hours after induction with arabinose. For the generation of positive controls, paraquat (PQ, 20 mM) was added to *E. coli* BW25113 pBAD33 pZE1-P*soxS*-GFP and H_2_O_2_ (100 μM) was added to *E. coli* BW25113 pBAD33 pZE1-P*dps*-GFP at the time of induction.

### ROS scavenging by chemical compounds

At the time of induction, DMSO (0.5% v/v), sodium pyruvate (10 mM), MnTBAP (100 μM) or a combination of these three were added. Alternatively, thiourea (150 mM) or 2,2′-dipyridyl (500 μM) were used. To examine the protective effect of DMSO, pyruvate and MnTBAP in the absence or presence of oxidative stress (Supplementary Fig. S1) the number of CFUs per ml was determined 1 hour after the addition of these compounds.

### Toxicity in anaerobic conditions

Anaerobic toxicity tests were performed in a Whitley DG250 anaerobic workstation (initial gas mixture comprised of 80% N_2_, 10% CO_2_, and 10% H_2_). All needed material, solutions and medium were placed under anaerobic conditions 24 hours prior to the experiment to remove oxygen. Overnight cultures were diluted under anaerobic conditions in LB medium with 40 mM NaNO_3_ as alternative electron acceptor, and grown without shaking at 37 °C until the OD_595 nm_ reached 0.4. Cultures were then induced with arabinose (0.2% w/v) and the number of CFUs per ml was determined at several time points before and after induction. Plates containing 40 mM NaNO_3_ were incubated overnight in anaerobic conditions.

### Statistics

All statistical analyses were performed with GraphPad Prism 6. Normality of representative data was verified by D’Agostino & Pearson omnibus normality test. Samples were compared by two tailed Student’s t tests.

## Additional Information

**How to cite this article**: Dewachter, L. *et al.* Reactive oxygen species do not contribute to ObgE*-mediated programmed cell death. *Sci. Rep.* **6**, 33723; doi: 10.1038/srep33723 (2016).

## Supplementary Material

Supplementary Information
