# Supplementary material for: Reactive oxygen species do not contribute to ObgE*-mediated programmed cell death
Source: Sci Rep. 2016 Sep 19;6:33723. doi: 10.1038/srep33723 (PMC5027388; doi:10.1038/srep33723)
Supplement: Supplementary Information [file srep33723-s1.pdf]

Supplementary Information

Reactive oxygen species do not contribute to ObgE*-mediated programmed cell death

Liselot Dewachter, Pauline Herpels, Natalie Verstraeten, Maarten Fauvart, Jan Michiels

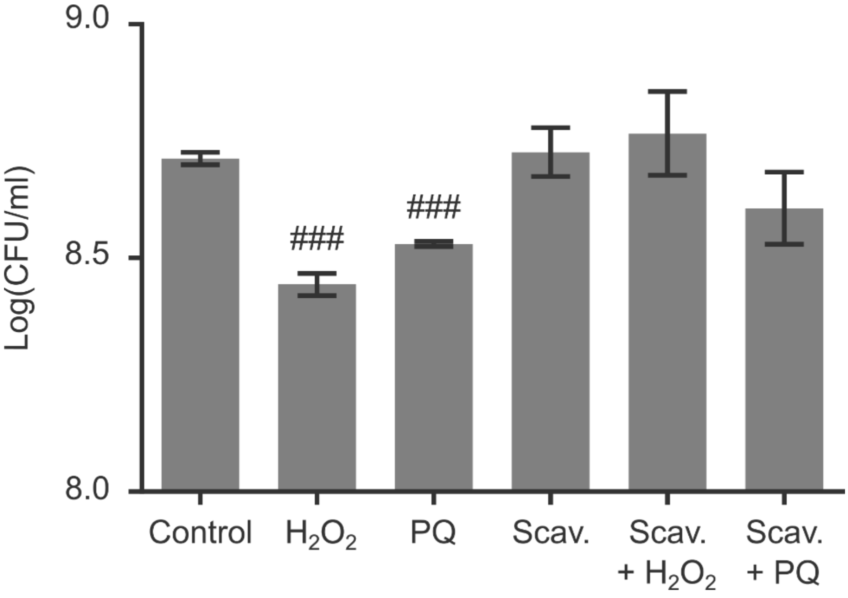

Supplementary Figure S1: The combination of selected scavengers increases survival in the presence of oxidative stress. Oxidative stress was imposed upon an exponential-phase culture of *E. coli* pBAD33 by adding 100 µM H_2O_2 or 20 mM paraquat (PQ). Survival was measured in the presence or absence of the combination of scavengers (Scav.); DMSO, pyruvate and MnTBAP. Error bars represent the standard error of the mean, n = 3. Student's t test: $^{###}$p-value < 0.001 in comparison with 'Control' (no scavengers and no oxidative stress).
